# Will the Doctor “See” You Now? The Development and Implementation of a Targeted Telemedicine System for Primary Care

**DOI:** 10.1055/s-0043-1776038

**Published:** 2023-10-18

**Authors:** Jeremy A. Epstein, Zoljargal Lkhagvajav, Tempest Young, Amanda Bertram, Hsin-Chieh Yeh, Casey Overby Taylor

**Affiliations:** 1 Division of General Internal Medicine, Johns Hopkins University School of Medicine, Baltimore, Maryland, United States; 2 Institute for Computational Medicine, Johns Hopkins University Whiting School of Engineering, Baltimore, Maryland, United States; 3 Department of Biomedical Engineering, Johns Hopkins University, Baltimore, Maryland, United States

**Keywords:** telemedicine, telehealth, underserved populations, primary Care, communication, patient satisfaction, communications

## Abstract

**Objectives:**

The coronavirus disease 2019 (COVID-19) pandemic led to a rapid adoption of telehealth. For underserved populations lacking internet access, telemedicine was accomplished by phone rather than an audio–video connection. The latter is presumed a more effective form and better approximation of an in-person visit. We sought to provide a telehealth platform to overcome barriers for underserved groups to hold video visits with their health care providers and evaluate differences between the two telehealth modalities as assessed by physicians and patients.

**Methods:**

We designed a simplified tablet solution for video visits and piloted its use among patients who otherwise would have been completing audio-only visits. Patients consented to participation and were randomized in a 1:1 fashion to continue with their scheduled phone visit (control) versus being shipped a tablet to facilitate a video visit (intervention). Participants and providers completed communication and satisfaction surveys.

**Results:**

Tablet and connectivity design features included removal of all functions but for the telemedicine program, LTE always-on wireless internet connectivity, absence of external equipment (cords chargers and keyboard), and no registration with a digital portal. In total, 18 patients were enrolled. Intervention patients with video-enabled devices compared to control patients agreed more strongly that they were satisfied with their visits (4.75/5 vs. 3.75/5, *p* = 0.02).

**Conclusion:**

The delivered simplified tablet solution for video visits holds promise to improve access to video visits for underserved groups. Strategies to facilitate patient acceptance of devices are needed to expand the scope and potential impact of this effort.

## Introduction

The coronavirus disease 2019 (COVID-19) pandemic led to a rapid adoption of telehealth. A recent report stated that nearly half (43.5%) of Medicare primary care visits were provided via telehealth in April 2020 as compared with 0.1% in February 2020.^1^ The report further states that the use of telehealth in primary care declined somewhat after in-person visits started to resume from mid-April through May 2020 but then appears to have leveled off at a persistent and significant level by June 2020. This trend suggests a continued interest in telehealth use for care delivery.

Prior to the pandemic, disparities in patient access to technology had relatively little impact on the provision of medical services, which were overwhelmingly delivered person-to-person at physical clinic sites. With the swift adoption of telehealth as the dominant form of outpatient medical care during the pandemic, however, the impact of disparities in technology access became more apparent.^2–4^ The ability to access and use smartphones or camera-outfitted internet-connected devices stratifies telehealth care delivery into two discrete categories—telephone and video.

The inability to access and use smartphones or camera-outfitted internet-connected devices during the COVID-19 pandemic relegates a group of largely economically disadvantaged patients to receive audio-only telemedicine.^5^ This raises the risk of widening healthcare inequities given the concern among medical providers that telephone-based care may be inferior to video-based care.^5,6^ The reasons for this belief are multifactorial. Firstly, visual input is essential in certain fields such as dermatology and neurology for accurate diagnosis.^7^ Secondly, being able to visualize patients may help with assessing their severity of illness and facilitating appropriate triage or avoidance of emergency care. Finally, under certain circumstances, video communication appears to confer benefits for patient–provider communication and relationship-building.^8–10^

A review of internal data from earlier in the transition to telemedicine at the Johns Hopkins Outpatient Center Internal Medicine Resident primary care clinic (3,409 total patients, 89 providers) showed that 86% of 550 telemedicine visits occurred by telephone rather than video. This occurred even though there was an enterprise-wide push toward video medicine. Prior to COVID-19, understanding the differences in the impact between telephone and video-based medicine simply had little relevance. Legal regulation limited the scope of telehealth services with telephone-based care to having only a supportive role, and video visits being largely limited to use in rural America to broaden access to specialized care.

Despite a common view that telephone-based care is inferior to video-based care, few high-quality studies have compared the impact of each modality on patient and provider satisfaction.^11,12^ One trial to date that randomized patients to audio versus video telemedicine found patient satisfaction to be higher with audio-only visits.^13^ Notably, the patients studied here differed from our population of interest in that they already had the capacity to perform video visits and were randomized to continue by this modality versus change to audio-only. Moreover, the clinical sites included specialty care. As we face a future with a strong telemedicine presence, we need to identify which patients cannot participate in video-based care and understand the differences between telephone and video telemedicine in terms of patient and provider satisfaction, as well as its impact on patient care.

## Objectives

The overall goal of this work is to pilot a framework for the set-up, delivery, and use of tablets by underserved patients for video-based telemedicine visits. Furthermore, we compared the video to telephone-based medicine in the primary care setting in terms of patient and provider attitudes: (1) perceived satisfaction with the telemedicine format; (2) ability to explain medical problems; and (3) feeling as satisfied as talking in person. In addition, we assessed patients’ perceived quality of their health care provider and the quality of the patient–provider interaction. Providers rated the perceived effectiveness of the telemedicine format on the clinical assessment and management of the patient. We hypothesized that patient and provider attitudes will be more positive with video-based telehealth when compared to phone-based telehealth.

## Methods

### Study Participants, Inclusion/Exclusion Criteria, and Enrollment

We contacted adult patients with a residential address in the state of Maryland and within a shipping radius that cost less than $20 and that were scheduled for a telephone-based visit. Patients with significant visual or hearing impairment, who have legal guardians or who were marked as lacking health care decision-making capacity, were excluded. In addition, we excluded patients who had not selected English as their preferred language. To find these patients, study group members manually reviewed the appointment notes of upcoming telemedicine visits at the Johns Hopkins Outpatient Center Internal Medicine Resident primary care clinic, excluding encounters that clearly indicated the patient’s capacity and plan for a video visit.

Next, eligible patients were contacted for further screening to ensure that they had a scheduled telephone-visit due to a lack of ability to have a video visit (i.e., no computer or smartphone with a camera and internet connectivity) and to obtain informed consent orally. Patients were enrolled in a 1:1 fashion into either the intervention arm or control arm. The control arm continued with their scheduled phone visits, per standard of care. Patients from both groups were provided gift cards in exchange for their participation. We enrolled patients between February 2021 and December 21. This study was performed in compliance with the World Medical Association Declaration of Helsinki on Ethical Principles for Medical Research Involving Human Subjects and approved by the Johns Hopkins University School of Medicine Institutional Review Board (reference no. 00257994).

### Mechanics of Delivery/Pick up

**Fig. 1:**
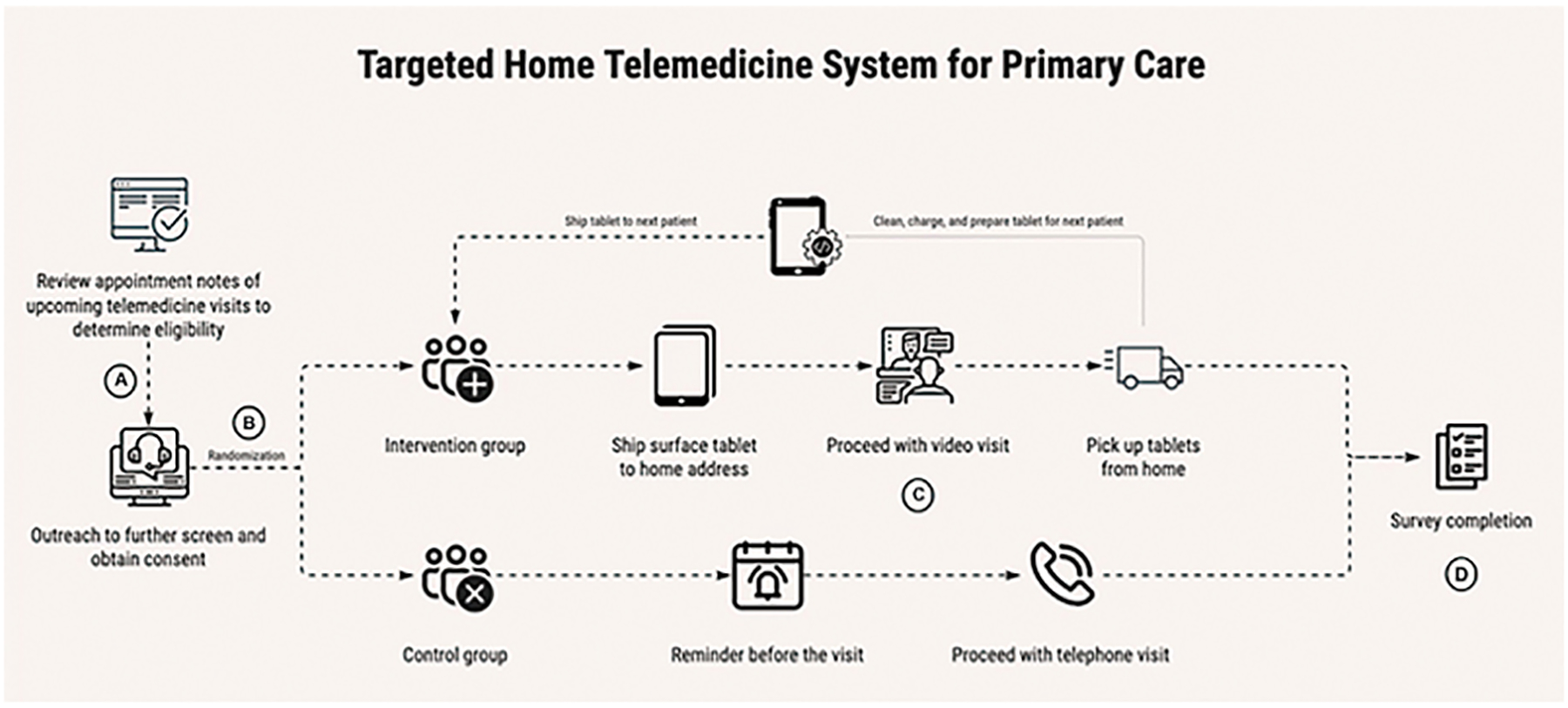
Targeted home telemedicine system for primary care—process for screening (A), randomization (B), visit completion (C), and survey completion (D).

This pilot project circulated six devices (Microsoft Surface Go tablets) to patients for video visit use. A device was shipped to the home address of each patient in the intervention arm in advance of the visit. Following the session, it was picked up from the patient’s home and shipped back to the clinic. The device was then cleaned, charged, and prepared for delivery to the next patient. Durable, waterproof, and well-cushioned electronics cases doubled as reusable shipping packaging, minimizing costs. The study team called the patient 24 to 72 hours prior to their visit to answer any questions they had about using the devices. Patients in the control group were similarly contacted 24 to 72 hours prior to their visit. This served to balance the amount of personal attention given to both groups of patients by study team members, minimizing a potential source of bias in which satisfaction and communication results may favor the intervention arm. This process is illustrated in Fig. 1.

### Simplified Device

**Fig. 2:**
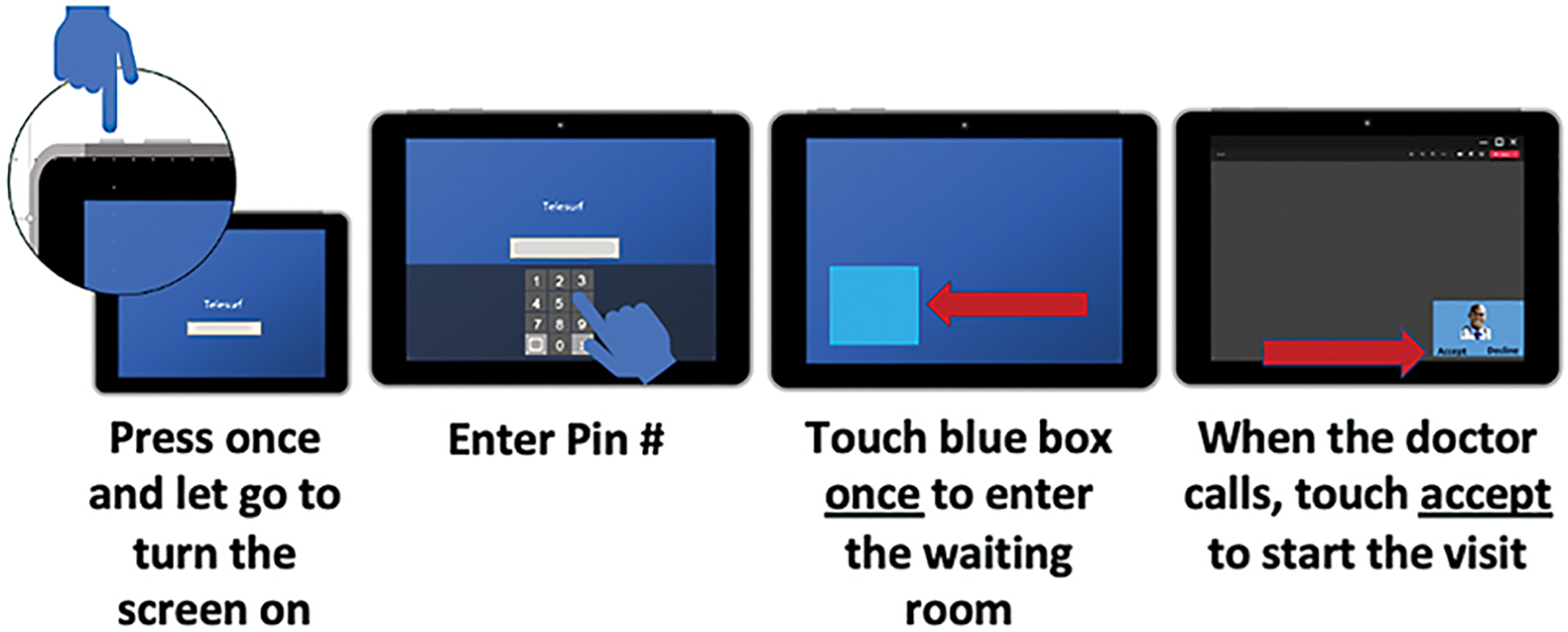
Video telemedicine process from the patient’s perspective.

Recognizing that our target population may be unfamiliar and potentially uncomfortable with computers, we worked to simplify device use as much as possible. In partnership with Microsoft, we customized the Surface Go tablet devices for this study. From the patient’s perspective, to use the device they turned it on, logged in with a PIN, and touched the desktop icon for Teams. Upon receiving a provider call, they clicked a pop-up window to “accept” and start the video visit. Fig. 2 illustrates this process. All other functions of the device were disabled and were not visible to the user. The device was accompanied by simple pictorial instructions to illustrate how to turn on the device and get set up for the video visit. The devices were precharged, and no cords were included. This process stood in contrast to the standard video visit procedure at the time that required patients to go through an initial registration process and then log in using an online portal to access their video visit. From the perspective of providers, rather than using the telemedicine platform that was embedded in the electronic health record (EHR), clinicians would directly dial the patient’s device using a U.S. Health Insurance Portability and Accountability Act-compliant, secure, and institutionally authorized platform, Microsoft Teams. At the time of this study, the Teams program was already in regular, daily, use as a part of the clinic workflow.

### Internet Delivery

One potential approach to expand access for patients to video-based telemedicine would have been to provide computers that could tap into existing WiFi networks. Free accessible public WiFi, however, was not available in the homes of patients in our catchment area. We considered that some patients may already have computers or phones with video capability but otherwise lack mobile phone data plans or a suitable internet connection. While the provision of just a mobile hotspot might have facilitated video visits for these patients, introducing heterogeneity in the intervention arm would have adversely impacted our ability to reach any conclusion about our study’s impact. We therefore equipped our devices with LTE connectivity and shipped them to patients’ homes. This ensured reliable internet connectivity and allowed patients to remain in their homes for their video-based telemedicine visits.

### Survey Design and Distribution

Patient and provider surveys were adapted from previously published instruments. Patients evaluated the quality of their provider using a revised Hospital Consumer Assessment of Healthcare Providers and Systems survey by Palen et al.^14^ Questions from the Perceptions of Satisfaction and Usefulness Questionnaire by Bakken et al were incorporated to assess patient and provider satisfaction and communication.^15^ Lastly, a survey by Glaser et al was adapted to gauge the clinical effectiveness of the visit as perceived by providers.^16^

To assess patients’ perceived quality of their health care provider when interacting by video versus telephone, patients in both groups were asked to rate the provider on a 0 to 10 scale where 0 is the worst provider possible and 10 is the best provider possible.

The quality of the provider interaction was assessed by four subscales: having clear explanations, feeling heard, feeling respected, and having enough time with the provider. Items were rated on a Yes/No scale: yes indicates feeling satisfied with the provider interaction.

The satisfaction with the telemedicine visit was assessed by three subscales: perceived satisfaction with the telemedicine format in general, ability to explain or understand medical problems well enough, and feeling as satisfied as talking to the doctor or patient in person. Items were rated on a 5-point Likert scale: higher point indicates higher satisfaction with the telemedicine visit. The effectiveness of the telemedicine format for the clinical assessment and management of the patient was evaluated by the providers using three subscales: perceived effectiveness of the telemedicine visit in general, the potential that an in-person visit would yield clinically relevant or management-changing information. The items were rated on a 5-point Likert scale: for the first subscale, a higher point indicates higher effectiveness of the telemedicine format while for the last two subscales, a higher point indicates lower effectiveness of the telemedicine format.

The patient survey further asked about education, perceived health status, and computer usage. The provider survey requested demographic information, postgraduate year, and why the telemedicine technology did not work if the visit was not completed.

In this pilot study, we offered multiple mechanisms to collect survey responses from patient study participants. For video visits, patient study participants could complete surveys in one of three ways: (1) by completing a web-based survey that appeared on the screen after the medical visit; (2) by completing a paper-based survey with a preaddressed and stamped return envelope; or (3) by completing the survey responses over the phone with a study team member. Instructions for the patient study participant to activate the web-based survey were provided with the tablet; however, if they forgot or chose not to complete the web-based survey, the paper-based survey was mailed to them by the study team. For phone visits, patient study participants could complete surveys one of two ways: (1) by completing a paper-based survey that was sent to them by mail; or (2) by completing the survey responses over the phone with a study team member.

### Data Analysis

Data analyses were conducted using STATA/BE version 17.0. Independent means *t*-tests and Fisher’s exact test compared intervention group participants to control group participants as well as acceptors to decliners to participate in the study. Free-text responses were summarized and used to provide context for our findings. Patient and provider attitudes were assessed using descriptive statistics. In addition, we used independent *t*-tests to compare the difference in rating of the patient and provider attitudes with and without device use for video visits. Statistical significance criterion was set at *p* < 0.05.

## Results

### Study Participants

**Fig. 3:**
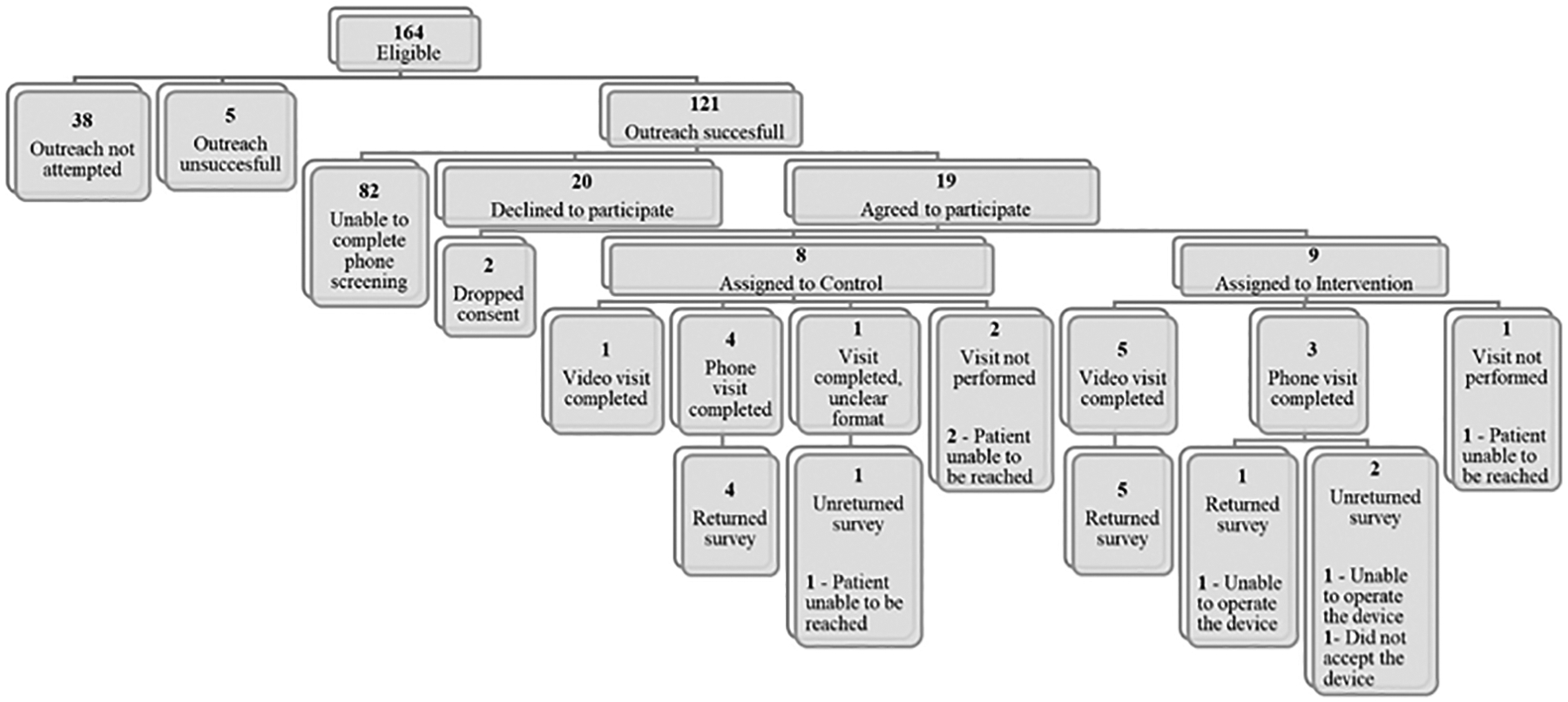
Flowchart showing patient recruitment, randomization, and visit and survey completion.

By reviewing the appointment notes, we identified 164 patients with upcoming visits that appeared to be by phone and who met eligibility criteria. Of the 121 patients successfully reached by the study team, 82 did not complete the phone screening protocol, and 20 declined to participate. In total, 19 patients consented to participation although two subsequently withdrew consent. Seventeen patients underwent randomization to the intervention group (nine patients) and control group (eight patients). Four patients from the control group completed a phone visit, while three did not complete their visits. One patient used their own video-enabled device to complete a video visit. From the intervention group, five patients completed a video visit and one patient could not use the device and completed a phone visit. Three patients did not complete the visit. Five patients who completed a phone visit and five patients who completed a video visit responded to the survey (Fig. 3).

The trial participants were a median age of 58 years, majority African American and not Hispanic or Latino, and had Medicare, Medicare Advantage, or Medicaid as their insurance (Table 1). The majority of the telemedicine visits were between patients and their own primary care doctors as opposed to being with covering physicians. There were no significant differences between groups except for gender. In total, 89% of participants were female in the intervention group versus 38% in the control group (*p*-value = 0.05).

### Characterization of Patients Who Declined Participation

Of the 20 individuals who declined to participate in the study, 14 provided explanations: nonspecific personal reasons (seven people), preference for phone over video (five people), not comfortable displaying themselves (one person), not comfortable using a computer (one person). The demographics of patients who declined participation were not different than those who accepted (Supplementary Appendix 1, available in the online version).

### Patient Satisfaction

Five patients who completed a phone visit and five patients who completed a video visit filled out the post-telemedicine-visit survey about their satisfaction. The perceived quality of the healthcare provider as well as the quality of the patient–provider interaction were universally high and there was no statistical significance between groups (Supplementary Appendix 2, available in the online version).

The mean score was statistically significantly higher in the video visit group than in the phone visit group in terms of all three subscales assessing telemedicine satisfaction (Table 2). Respondents from the video visit group were more satisfied with the telemedicine modality they used for the visit, in general. Their mean score for the question, if they could explain their medical problems well enough, was statistically significantly higher. They had a statistically significantly higher mean score in the question evaluating if talking to the doctor using the telemedicine modality was as satisfying as talking in person (*p* = 0.008, *p* = 0.04, and *p* = 0.028, respectively).

### Patient Health, Education, and Technology Assessments

There were no statistically significant differences between study groups in their self-assessments of health, education, and technology use (Supplementary Appendix 3, available in the online version). Patients generally rated their physical and mental health as good. Education levels ranged from having a partial high school education to having completed some college education or a 2-year degree. As expected, a majority of patients were not using computers at home. Cost and lack of familiarity with the technology were the main barriers to smartphone and internet/computer use (Supplementary Appendix 4, available in the online version).

### Effectiveness of the Telemedicine Formats

Four providers returned survey data. Three had completed video visits and one completed a phone visit. There were no statistically significant differences in any measure of satisfaction or efficacy between the providers who provided video visits and those who provided phone visits (Supplementary Appendix 5, available in the online version).

## Discussion

Our study describes a new framework to facilitate video-based telemedicine visits via the delivery of customized easy-to-use internet-connected tablets for patients who otherwise lacked the requisite technological capacity. By randomly assigning patients to either receive a device versus continue with their upcoming telephone-only visit, we additionally collected high-quality data comparing these two modalities of telemedicine visits.

### Summary of Main Findings and Outcomes

In summary, we designed a tablet solution for ease of use and successfully piloted our framework for the set-up, delivery, and use of those tablets by patients who previously were unable to complete video-based telemedicine visits. In addition, our findings indicated that regardless of modality patient satisfaction was high (mean of 3.6 or more for all questions, Table 2). While it may not be surprising to see higher satisfaction among those completing video-based visits, when compared to those completing audio-based visits among a sample of patients with technology access issues, this study provides some additional insight into the magnitude of benefit that could be achieved with improved technology access. Three items, however, were rated more favorably by patients who completed video visits. These included satisfaction with the visit modality, satisfaction with talking to their doctor, and their capacity to explain medical issues (*p* = 0.008, *p* = 0.04, *p* = 0.028, respectively). This finding occurred despite limited enrollment. This study also identified several lessons from our experiences with identifying eligible patients and from implementing our model to make telehealth more accessible.

### Lessons Learned from Identifying and Enrolling Underserved Patients

The predominant barrier we encountered to increasing distribution of the telemedicine devices was low patient enrollment. This stemmed from difficulty in both finding and recruiting patients. As there was no EHR field designating which patients lacked the capacity to conduct video visits, manual review was needed. This time-intensive process involved screening 750 patient appointment scheduling notes, yielding 164 potentially eligible patients. Patient engagement in the study enrollment process was also limiting. Of these, 164 potentially eligible patients, only 19 agreed to participate. The reasons potential participants declined enrollment were often unable to be determined as a large majority of patients prematurely ended the calls made by the research team or did not provide any reason for their disinterest. Potential explanations for this behavior include suspicion of medical research and a complicated protracted enrollmentscript required by the institutional review board. For the small numbers who provided a rationale fordeclining study participation, excluding thosewithpersonal circumstances, patients primarily stated that it was either their preference for phone or their discomfort with the video that drove disinterest in the study.

Recognizing the limitations associated with EHR data, our screening process from the onset, involved talking directly with patients. Even with this robust screening protocol, however, several patients ultimately were selected for participation who did not meet prespecified inclusion and exclusion criteria. Two of the nine patients assigned to the tablet device were subsequently found to have significant cognitive impairment and psychiatric disease that rendered them unable or unwilling to participate. In the control arm, one of eight patients was able to procure a video device although their inclusion in the study was predicated on their inability to do so.

Based on billing data that distinguish video versus audio-only telemedicine, a large number of patients without the capacity to perform video telemedicine were seemingly not being reached by our efforts. Some of these visits may have taken place via audio-only connections for other reasons—technical failure of the video client, provider-side issues, patient refusal, or patient error despite their theoretical capacity. With such a high drop-off because of patients not being reached for consent, we presume that this played a large role in our recruitment numbers. Two potential avenues for increasing patient access to devices include (1) orienting patients to these devices during in-person visits to alleviate any concerns and increase their acceptance and (2) changing workflow so that patients are identified and screened to receive a device while a future visit is being scheduled, thereby eliminating the need to subsequently connect with and consent patients by phone call.

### Lessons Learned from the Pilot

We found that for those patients who agreed to participate and were randomized to the intervention arm, device delivery before the visit and retrieval after the visit using a standard domestic shipping company was an efficient and effective process. This allowed a small number of internet-enabled devices to reach large numbers of patients. Two patients were unable to access the telemedicine platform even though they were using the devices appropriately and had live support from research team personnel. Although the computers were formatted to be easier to operate, the first version of our modified platform contained a glitch that could lead to a failure of the video platform to initialize. Subsequent iterations of our devices eliminated this problem. Were it not for these technical issues with our platform, seven of nine patients in the intervention arm would have completed video-based telemedicine visits. This is similar to the 82% of tablet use by patients who received devices through the VA as part of an effort to improve rural telemedicine.^17^

While distributing computers into the community may raise concerns about device damage or loss, there were no instances of either throughout our trial. Location tracking software was installed on all devices as a deterrent and this was communicated through a sticker placed on all tablets. The software was never used. On two occasions, research personnel presented with permission to patients’ homes for device retrieval, but this was due to participant confusion about the return process. Other studies involving the distribution of telehealth technology have had loss rates of 20 to 30%.^18^ Our low rate of loss may be a product of a small sample size, however.

### Limitations

While this pilot project demonstrates both the feasibility and promise of this approach to telemedicine delivery, certain limitations should be considered. Given the trial was conducted for a single clinic within one health system, our findings may not be generalizable to other practices. While our implementation was with one site, the tools used for our implementation are employed across the country—teleconferencing software (Microsoft Teams) and EHR (EPIC). Adjustment of this care delivery model would need to take into account local factors including the location and geographic distribution of patient homes and the existing telemedicine process and scheduling practices. Our model required additional resources to implement, including internet data plans, tablets, shipping costs, and time from research study personnel. While these costs may be offset by increased reimbursement for video-based telemedicine, further exploration of this telemedicine model’s financial impact is needed. Our exclusion of non-English-speaking patients, a functional necessity due to operational constraints of our pilot project, may have introduced an unmeasured selection bias. Both to avoid exacerbating any underlying inequity in video-based telemedicine access and to broaden the study recruitment pool, efforts should be made to include this patient population in the future.

## Conclusion

This study shows promise for the feasibility of facilitating video-based telemedicine primary care visits for patients with limited technological literacy through the delivery of simplified internet-connected devices. Data from this pilot project support the widely held belief that video-based telemedicine is superior to phone-based telemedicine in terms of patient satisfaction and communication. Further, larger-scale research is needed to be able to evaluate differences between audio and video telemedicine. Our outreach model relied on reaching out to patients after telemedicine visits had been scheduled. Embedding within the scheduling process an assessment of capacity for video-based telemedicine with the option to request a device would be an appealing strategy to expand device deployment. A large segment of the patient population prefers phone over video for the purposes of telemedicine. Without further understanding the rationale behind this sentiment, any intervention that simply provides the capacity to conduct video-based telemedicine will not be effective at transforming audio to video-based visits. Given diminishing reimbursement for audio-only visits versus audio–video visits, broadening access to video-based telemedicine through this process may not only reduce inequities in health care access but also be cost-neutral or even financially advantageous.^19^

## Supplementary Material

Appendix

## Figures and Tables

**Table 1:** Demographics, insurance coverage, and telemedicine visit providers of study participants

	Patients			
	Total (*n* = 17)	Intervention (*n* = 9)	Control (*n* = 8)	*p*-Value
Age (years)—median (IQR)	57 (49–61)	61 (55–74)	49.5 (41–59)	0.1
Sex—*n* (%)				0.05
Male	6 (35.3)	1 (11.1)	5 (62.5)	
Female	11 (64.7)	8 (89.9)	3 (37.5)	
Race & ethnicity—*n* (%)				0.2
African American and Hispanic or Latino	1 (5.9)	0	1 (12.5)	
African American and not Hispanic or Latino	11 (64.7)	5 (55.6)	6 (75)	
White or Caucasian and not Hispanic or Latino	3 (17.6)	3 (33.3)	0	
Other race and not Hispanic or Latino	2 (11.8)	1 (11.1)	1 (12.5)	
Telemedicine visit provider—*n* (%)				0.6
Listed primary care provide	11 (64.7)	5 (55.6)	6 (75)	
Covering provider	6 (35.3)	4 (44.4)	2 (25)	
Primary coverage—*n* (%)				0.8
Medicare	6 (35.3)	3 (33.3)	3 (37.5)	
Medicaid	5 (29.4)	2 (22.2)	3 (37.5)	
Medicare advantage	4 (23.5)	3 (33.3)	1 (12.5)	
Commercial	2 (11.8)	1 (11.1)	1 (12.5)	

Abbreviation: IQR, interquartile range.

**Table 2:** Patient survey results—provider quality, communication ability, and satisfaction with telemedicine visit (video visit group vs. phone visit group)

	Patients		
	Video visit (*n* = 5)	Phone visit (*n* = 5)	*p*-Value
Using any number from 0 to 10, where 0 is the worst provider possible and 10 is the best provider possible, what number would you use to rate this provider (mean score)?	9.8	9.2	0.14
I could explain my medical problems well enough (mean score)	4.6	4	0.04
In general, I was satisfied with using a video or phone call for this visit (mean score)	4.8	3.8	0.008
Talking to the doctor was as satisfying as talking in person (mean score)	4.8	3.6	0.028

## Clinical Relevance Statement

Large numbers of patients remain unable to participate in video-based telemedicine. One mechanism to expand video telemedicine access is to deliver easy-to-use internet-connected computers to patients in need and retrieve them after the visit. This as-needed model for video telemedicine allows for a small number of devices to serve the telemedicine needs of a large clinic population.
