# Supplementary material for: Will the Doctor “See” You Now? The Development and Implementation of a Targeted Telemedicine System for Primary Care
Source: ACI open. Author manuscript; Available in PMC 2023 Oct 27. (PMC10610031; doi:10.1055/s-0043-1776038)
Supplement: Appendix [file NIHMS1938857-supplement-Appendix.docx]

*Supplementary Appendix 1. Acceptors and decliners of the study participation*

|  | **Patients** | | | |  |
| --- | --- | --- | --- | --- | --- |
|  | **Total** | **Acceptors** | **Decliners** | p | |
|  | (n=39) | (n=19) | (n=20) | value | |
| **Age (years) – Median (IQR)** | 57 (42-64) | 57 (42-61) | 56.5 (41-68) | 1.0 | |
| **Sex - n (%)** |  |  |  | 0.82 | |
| Male | 13 (33.6) | 6 (31.5) | 7 (35) |  | |
| Female | 26 (66.7) | 13 (68.4) | 13 (65) |  | |
| **Race & Ethnicity n (%)** |  |  |  | 0.16 | |
| African American & Hispanic or Latino White | 1 (2.6) | 1 (5.3) | 0 |  | |
| African American & Not     Hispanic or Latino | 32 (82.1) | 13 (68.4) | 19 (95) |  | |
| White or Caucasian & Not Hispanic or Latino African American | 4 (10.3) | 3 (15.8) | 1 (5) |  | |
| Other race & Not Hispanic or Latino | 2 (5.1) | 2 (10.5) | 0 |  | |
| **Telemedicine visit provider** |  |  |  | 0.42 | |
| Listed PCP | 20 (51.3) | 11 (57.9) | 9 (45) |  | |
| Covering provider | 19 (48.7) | 8 (42.1) | 11 (55) |  | |
| **Primary Coverage** |  |  |  | 0.18 | |
| Medicare | 14 (35.9) | 6 (31.6) | 8 (40) |  | |
| Medicaid | 13 (33.3) | 6 (31.6) | 7 (35) |  | |
| Medicare Advantage | 4 (10.3) | 4 (21) | 0 |  | |
| Commercial | 8 (20.5) | 3 (15.8) | 5 (25) |  | |
| Abbreviation: IQR, interquartile range |  |  |  |  | |

*Supplementary Appendix 2. Patient survey results, quality of provider interaction (video visit group vs phone visit group)*

|  | **Patients** | |
| --- | --- | --- |
|  | **Video visit** | **Phone visit** |
|  | (n=5) | (n=5) |
| Did this provider explain things in a way that was easy to understand? (Yes %) | 100% | 100% |
| Did this provider listen carefully to you? (Yes %) | 100% | 100% |
| Did this provider show respect for what you had to say? (Yes %) | 100% | 100% |
| Did this provider spend enough time with you? (Yes %) | 100% | 100% |

*Supplementary Appendix 3. Patient health and education survey results (video visit group vs phone visit group)*

|  | **Patients** | | |
| --- | --- | --- | --- |
|  | **Video visit** | **Phone visit** | p |
|  | (n=5) | (n=5) | value |
| In general, how would you rate your overall health? (Mean score) | 3.4 | 3.4 | 1.00 |
| In general, how would you rate your mental or emotional health? (Mean score) | 3.4 | 3.2 | 0.67 |
| What is the highest grade or level of school that you have completed? (n, %) |  |  | 1.0 |
| 8^th^ grade or less | - | - |  |
| Some high school, but did not graduate | 1 (20%) | 1 (20%) |  |
| High school graduate or GED | 1 (20%) | 1 (20%) |  |
| Some college or 2-year degree | 3 (60%) | 3 (60%) |  |
| 4-year college graduate | - | - |  |
| More than a 4-year college degree | - | - |  |

*Supplementary Appendix 4. Patient computer utilization survey results (video visit group vs phone visit group)*

|  | **Patients** | | |
| --- | --- | --- | --- |
|  | **Video visit** | **Phone visit** | p |
|  | (n=5) | (n=5) | value |
| What is the main reason you do not have a phone with the internet or a computer with the internet? |  |  |  |
| Cost | 2 (40%) | 1 (20%) | 0.49 |
| Space | - | - |  |
| Do not feel that I need it | - | 1 (20%) | 0.29 |
| Would not know how to use it | 3 (60%) | 1 (20%) | 0.20 |
| Other | 1 (20%) | 1 (20%) | 1.0 |
| Have you ever used a computer? (Yes %) | 40% | 60% | 0.76 |
| Do you currently have a computer in your home or a computer that you use at home? (Yes %) | 25% | 40% | 0.64 |
| Do you use a computer at home at least once a week? (Yes %) | 20% | 20% | 1.0 |
| Do you have access to the internet or e-mail on your computer at home? (Yes %) | 40% | 40% | 1.0 |
| Do you use a computer to get health or medical information? (Yes %) | 20% | 20% | 1.0 |

*Supplementary Appendix 5. Provider perceived effectiveness survey results (video visit group vs phone visit group)*

|  | **Providers** | |
| --- | --- | --- |
|  | **Video visit** | **Phone visit** |
|  | (n=3) | (n=1) |
| In general, I was satisfied with using telemedicine for this visit (Mean score) | 4 | 4 |
| I was able to understand the patient's explanations of their medical problems well enough. If the patient had no medical problems this visit, leave blank (mean score) | 5 | 2 |
| In general, this telemedicine visit was effective in improving this patient's medical care (mean score) | 4.6 | 4 |
| An in-person visit would likely have provided additional  clinically relevant information (mean score) | 3.6 | 5 |
| An in-person visit would likely have led to a change in management (mean score) | 3 | 3 |
| Talking to the patient was as satisfying as talking in person. (mean score) | 3.3 | 2 |
